# Preparative Isolation of Three Anthraquinones from *Rumex japonicus* by High-Speed Counter-Current Chromatography 

**DOI:** 10.3390/molecules16021201

**Published:** 2011-01-27

**Authors:** Shuying Guo, Bo Feng, Ruonan Zhu, Jiankang Ma, Wei Wang

**Affiliations:** 1 College of Pharmacy, Jilin Medical College, Jilin 132013, China; E-Mails: 568823651@qq.com (S.G.); fengbo2@sina.com (B.F.); 2 Institute of Phytochemistry, Jilin Academy of Chinese Medicine Sciences, Changchun 130012, China; E-Mail: zhuruonan2008@yahoo.com.cn (R.Z.)

**Keywords:** *Rumex japonicus* Houtt., anthraquinones, high-speed counter-current chromatography (HSCCC), emodin, chrysophanol, physcion

## Abstract

Three anthraquinones—emodin, chrysophanol, and physcion—were successfully purified from the dichloromethane extract of the Chinese medicinal herb *Rumex japonicus* by high-speed counter-current chromatography (HSCCC). The extract was separated with *n*-hexane–ethanol–water (18:22:3, v/v/v) as the two-phase solvent system and yielded 3.4 mg of emodin, 24.1 mg of chrysophanol, and 2.0 mg of physcion from 500 mg of sample with purities of 99.2 %, 98.8% and 98.2%, respectively. The HSCCC fractions were analyzed by high-performance liquid chromatography (HPLC) and the chemical structures of the three anthraquinones were confirmed by ^1^H-NMR and ^13^C-NMR analysis. This is the first time these anthraquinones have been obtained from *R*.* japonicus* by HSCCC.

## 1. Introduction

**Figure 1:**
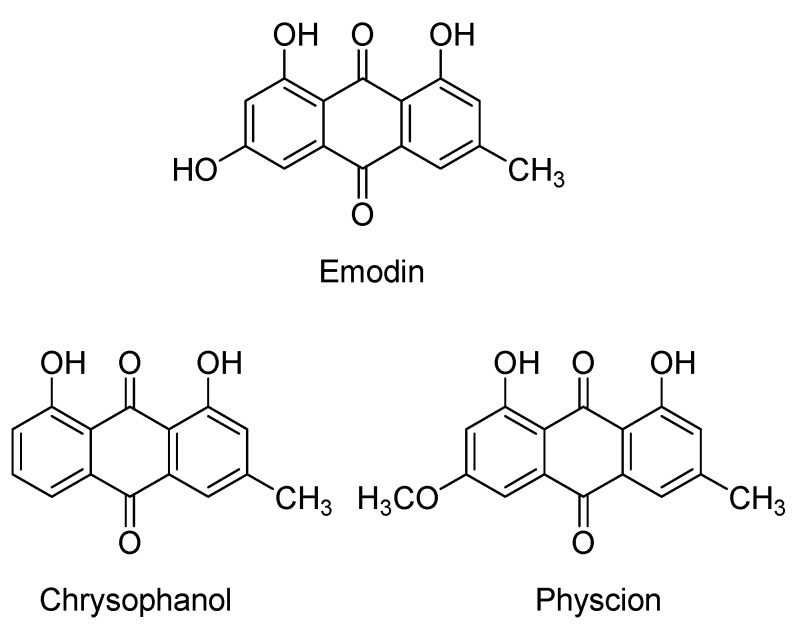
Chemical structures of the target compounds from *R. japonicas*.

Anthraquinones constitute an important class of natural compounds with widespread distribution and a wide range of activity. Every year a large number of anthraquinones, having varied substitution patterns, are isolated from Nature [1,2,3]. *Rumex japonicus* Houtt., a rich source of anthraquinones, has been traditionally used for the treatment of heat phlegm, jaundice, constipation, scabies, and uterine hemorrhage in East Asian countries such as China, Korea, and Japan [4]. The major anthraquinones of *R* .*japonicus*, including emodin, chrysophanol, and physcion (Figure 1), each have specific pharmaceutical activity. The most abundant anthraquinone of *R*. *japonicus*, chrysophanol, displays anti-inflammatory [5], anti-bacterial [6], hepatoprotective [7], and human protein tyrosine phosphatase 1B (hPTP1B) inhibiting effects [8]. The anti-inflammatory capability was reported to act through suppression of the activation of nuclear factor-κB (NF-κB) and caspase-1 [5]. Emodin is another major anthraquinone in *R*. *japonicus* found to possess protective effects on hepatocytes and cholangiocytes [7], anti-cancer properties [9], anti-inflammatory activity [10], anti-angiogenic activity [11], and inhibitory activities on protein glycation and aldose reductase [12]. Physcion is the other major anthraquinone in *R*. *japonicus* shown to have a number biological effects, including hepatoprotective [7], anti-inflammatory [10], anti-bacterial, and anti-fungal ones [13]. Emodin, chrysophanol, and physcion of high purity are needed for the quality control of products from *R*.*japonicus* or other related products, so it was deemed important to develop a method for the isolation and purification of all these compounds. 

The preparative isolation and purification of anthraquinones from plant materials by conventional methods such as macroporous resins, silica gel, RP-18 reversed-phase silica gel, Sephadex LH-20, and high-performance liquid chromatography have been reported previously. But these methods are tedious, time consuming, and have the peril of loss of compounds due to the highly adsorptive effects of the solid matrices. Therefore more efficient separation methods need to be explored further. High-speed counter-current chromatography (HSCCC) is a kind of support-free liquid-liquid partition chromatographic technique which was first invented by Ito [14] that can eliminate the irreversible adsorption of samples on solid support seen in conventional column chromatography. The method permits the introduction of crude samples into the column without extensive preparation, and has been successfully applied to isolate and purify a number of natural products, such as flavonoids [15,16], alkaloids [17,18], saponins [19,20], anthraquinones [21,22], and coumarins [23]. The separation and purification of emodin, chrysophanol, and physcion from the Chinese medical herb *Rheum officinale* Baill. by HSCCC have been reported previously [24,25,26]. In this paper, the two-phase solvent system composed of *n*-hexane–ethanol–water (18:22:3, v/v/v) was applied for the first time to the separation and purification of three anthraquinones – emodin, chrysophanol, and physcion – from *R*.* japonicus* by HSCCC. The purities of emodin, chrysophanol, and physcion were 99.2 %, 98.8% and 98.2%, respectively, as determined by HPLC. The chemical structures of the three target compounds were verified by ^1^H-NMR and ^13^C-NMR analysis.

## 2. Results and Discussion

### 2.1. HPLC analysis of the crude extract and HSCCC peak fractions

**Figure 2:**
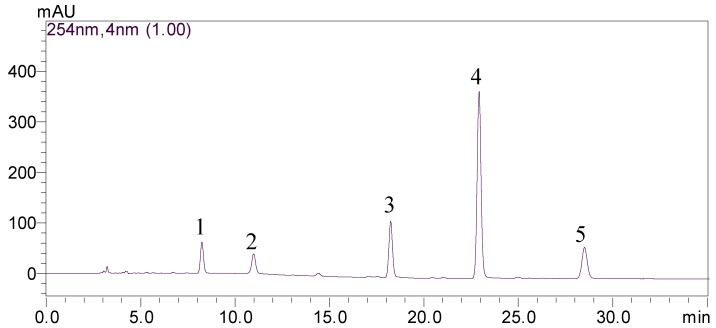
HPLC chromatogram of the crude extract from *R. japonicus*.

The crude extract and the fractions obtained by HSCCC were analyzed by HPLC. The analyses required the development of good HPLC separation conditions for each of the various components. In order to select an appropriate elution system for HPLC separation of sample, different kind of mobile phases, including methanol and phosphate buffer solution (pH 2.0), acetonitrile and phosphate buffer solution (pH 2.0), methanol and 0.1 % phosphoric acid solution, acetonitrile and 0.1 % phosphoric acid solution, methanol and 0.5 % acetic acid solution, and acetonitrile and 0.5 % acetic acid solution, were evaluated to find the best separation conditions. The optimum mobile phase was found to be a gradient prepared from methanol (A) and phosphate buffer solution (pH 2.0, B). A gradient program was used as follows: 0–6 min, isocratic elution with A-B (75:25, v/v); 6–17 min, linear change from A-B (75:25, v/v) to A-B (85:15, v/v); 17–30 min, isocratic elution with A-B (85:15, v/v); 30–35 min, linear change from A-B (85:15, v/v) to A-B (75:25, v/v). The total run time was 35 min and the column was reequilibrated for 10 min between runs. The flow rate was 1.0 mL/min and the column temperature was maintain at 30 °C. Detection was by monitoring of UV absorption at 254 nm. The crude extraxt and peak fractions separated by HSCCC were analyzed by HPLC under the optimum analytical conditions. The HPLC chromatogram of the crude extract is shown in Figure 2. Peaks 1–5 correspond to aloe-emodin, rhein, emodin, chrysophanol, and physcion.

### 2.2. Selection of two-phase solvent system for HSCCC

Successful separation by HSCCC depends upon the selection of a suitable two-phase solvent system, which provides an ideal range of the partition coefficient (*K*) for the targeted compounds. Several two-phase solvent systems were tested and the *K* values were measured and summarized in Table 1. The result of this experiment shows that the *K* value of emodin could be increased by increasing the volume ratio of ethanol. When the volume ratio of ethanol reaches 24, the two-phase of solvent system becomes miscible. Therefore, the two-phase solvent system composed of *n*-hexane–ethanol–water (18:22:3, v/v/v) was selected to isolate and purify the target compounds in the present paper. The *K* values of emodin, chrysophanol, and physcion with this phase system were 0.27, 1.19, and 0.91, respectively.

**Table 1:** *K* values of the target compounds in several two-phase solvent systems.

Solvent system ( *n*-hexane–ethanol–water) ( v/v/v)	*K* value		
	Fraction I	Fraction II	Fraction III
18:10:3	0.09	2.46	2.34
18:12:3	0.03	2.48	2.25
18:14:3	0.12	2.00	1.73
18:16:3	0.18	1.94	1.79
18:18:3	0.15	1.53	1.29
18:20:3	0.23	1.28	1.25
18:22:3	0.27	1.19	0.91

**Figure 3:**
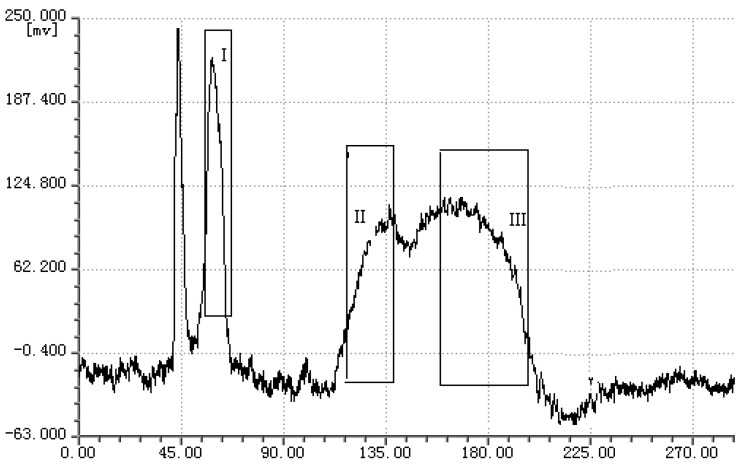
HSCCC chromatography of the crude extract from *R*. *japonicus*.

**Figure 4:**
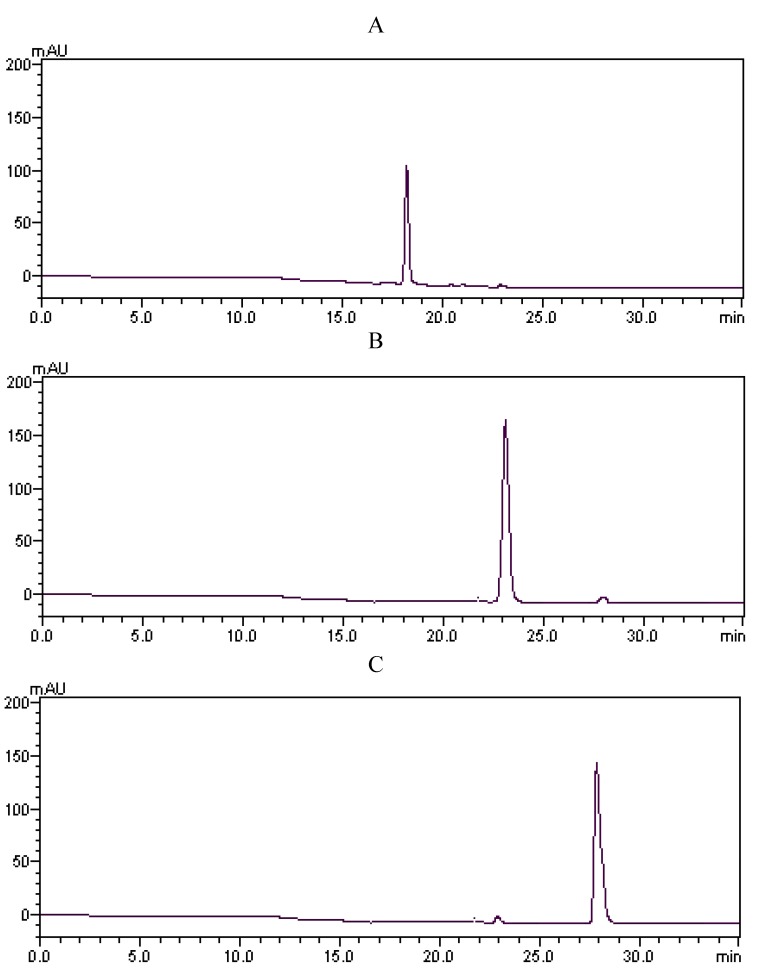
HPLC chromatograms of the HSCCC fractions. A: emodin from HSCCC fraction I; B: chrysophanol from HSCCC fraction II, and C: physcion from HSCCC fraction III.

Under the optimized conditions, three fractions (I, II, and III) were obtained in a one step elution and less than 5 h, namely 3.4 mg of fraction I (collected during 55–65 min), 24.1 mg of fraction II (collected during 120–140 min), and 2.0 mg of fraction III (collected during 160–195 min). The typical HSCCC chromatogram is shown in Figure 3. As shown in Figure 4, the HPLC analysis of each HSCCC fractions revealed that three pure anthraquinones could be obtained from the crude extract in one step. 

## 3. Experimental

### 3.1. Apparatus

The HSCCC instrument employed is a model TBE-300B high-speed counter-current chromatography (Shanghai Tauto Biotech Co. Ltd., Shanghai, China) with three multilayer coil separation column connected in series (I.D. of the tubing = 2.6 mm, total volume = 300 mL) and a 20 mL sample loop. The revolution radius or the distance between the holder axis and central axis of the centrifuge (*R*) was 5 cm, and the *β* values of the multilayer coil varied from 0.5 at internal terminal to 0.8 at the external terminal (*β* = *r*/*R*, where *r* is the distance from the coil to the holder shaft). The revolution speed of the apparatus can be regulated with a speed controller in the range between 0 and 1,000 rpm. The HSCCC system was equipped with a TBP-5002 constant flow pump and a TBD-2000 UV detector operating at 254 nm (Shanghai Tauto Biotech Co. Ltd., Shanghai, China), and a WH500-USB workstation (Shanghai Wuhao Information Technology Co. Ltd., China). An HX 1050 constant-temperature circulating implement (Beijing Boyikang Lab Instrument Company, Beijing, China) was used to control the separation temperature. 

The HPLC analyses were performed using a Shimadzu liquid chromatographic system (Shimadzu, Kyoto, Japan) equipped with LC-20AT quaternary solvent delivery system, an on-line degasser, SIL-20A auto-sampler, CTO-20A column temperature controller and SPD-M20A photodiode-array detector coupled with an analytical workstation.

The nuclear magnetic resonance (NMR) spectra were recorded on a Bruker AV-400 FT-NMR (^1^H at 400 MHz and ^13^C at 100 MHz) spectrometers. The EI-MS were obtained using a JEOL JMS mass spectrometer.

### 3.2. Reagents and materials

HPLC grade methanol (Merck, Darmstadt, Germany) and deionized water obtained from a Milli-Q system (Millipore, Bedford, MA, USA) were used for preparation of mobile phase. All solvents used for preparation of crude sample and HSCCC separation were of analytical grade (Beijing Chemical Engineering Factory, Beijing, China). The roots of *R*. *japonicus* were collected from Panshi county, Jilin Province of China, and authenticated by Prof. Jinghua Li, College of Pharmacy, Jilin Medical College, China. The corresponding voucher specimens are deposited at the Institute of Phytochemistry, Jilin Academy of Chinese Medicine Sciences, China.

### 3.3. Preparation of the crude sample

The roots of *R*. *japonicus* were pulverized and dried to constant weight before use. Approximately 5.0 g sample power was accurately weighed and transferred into a 50 mL centrifuge tube. Sample was ultrasonically extracted twice with 50 mL dichloromethane in a KQ-250DE apparatus (Kunshan Ultrasonic, KunShan, China) for 30 min at room temperature. The extracts were combined and evaporated to dryness, which yielded 0.74 g of crude extract. 

### 3.4. Selection of the two-phase solvent system

The composition of the two-phase solvent system was selected according to the partition coefficient (*K*) of target compounds of crude example. The partition coefficients were determined by HPLC as follows: a suitable amount of crude example was dissolved in 2 mL of aqueous phase of the pre-equilibrated two-phase solvent system. The solution was determined by HPLC and the peak area was recorded as *A*1. Then equal volume of the organic phase was added to the solution and mixed thoroughly. After the equilibration was established, the aqueous phase was determined by HPLC again and the peak area was recorded as *A*2. The partition coefficient (*K*) was obtained by the following equation: *K*= (*A*1 −*A*2)/*A*2.

### 3.5. Preparation of the two-phase solvent system and sample solution

The two-phase solvent system composed of *n*-hexane–ethanol–water (18:22:3, v/v/v) was used for HSCCC separation. It was prepared by adding the solvent to a separation funnel according to the volume ratios and thoroughly equilibrated by shaking repeatedly. Then, the upper phase and the lower phase were separated and degassed by sonication for 30 min shortly before use. The sample solution was prepared by dissolving 500 mg of the crude extract in 10 mL of the lower phase and 10 mL of the upper phase of *n*-hexane–ethanol–water (18:22:3, v/v/v).

### 3.6. HSCCC separation

In HSCCC separation, the coil column was first entirely filled with the upper phase of the solvent system. Then the apparatus was rotated at 850 rpm, while the lower phase was pumped into the column at flow rate of 2.0 mL/min. After the mobile phase front emerged and hydrodynamic equilibrium was reached in the column, 20 mL sample solution was injected through the injection valve. The effluent from the outlet of the column was continuously monitored with a UV detector at 254 nm. The temperature of the apparatus was set at 25 °C. Each peak fraction was manually collected following the chromatogram of each component.

### 3.7. HPLC analysis and identification of HSCCC peak fractions

The crude sample and each HSCCC peak fraction were analyzed by HPLC. The column was a Diamonsil ODS column (5 *μ*m, 250 mm × 4.6 mm, Dikma Technologies, Beijing, China), at a column temperature 30 °C. The mobile phase consisted of methanol (A) and phosphate buffer solution (pH 2.0, B). A gradient program was used as follows: 0–6 min, isocratic elution with A-B (75:25, v/v); 6–17 min, linear change from A-B (75:25, v/v) to A-B (85:15, v/v); 17–30 min, isocratic elution with A-B (85:15, v/v); 30–35 min, linear change from A-B (85:15, v/v) to A-B (75:25, v/v). The flow rate was 1.0 mL/min, and the detection was monitored at 254 nm. Identification of the HSCCC fractions was performed by ^1^H-NMR and ^13^C-NMR.

### 3.8. The structural identification

The chemical structure of each fraction of HSCCC was identified according to its EI-MS, ^1^H-NMR and ^13^C-NMR data.

Fraction I: EI-MS *m/z*: 270 [M]^+^; ^1^H-NMR (400 MHz, DMSO-*d*_6_) δ: 12.07 (1H, s, -OH), 12.00 (1H, s, -OH), 11.35 (1H, br s, -OH), 7.48 (1H, d, *J* = 1.2 Hz, H-4), 7.15 (1H, d, *J* = 1.2 Hz, H-2), 7.11 (1H, d, *J* = 2.0 Hz, H-5), 6.59 (1H, d, *J* = 2.0 Hz, H-7), 2.38 (3H, s, CH_3_); ^13^C-NMR (100 MHz, DMSO-*d*_6_) δ: 189.4 (C-9), 181.0 (C-10), 165.2 (C-8), 164.1 (C-1), 161.1 (C-6), 148.0 (C-3), 134.9 (C-10a), 132.6 (C-4a), 123.9 (C-4), 120.2 (C-2), 113.1(C-9a), 108.7 (C-5), 108.5(C-8a), 107.7(C-7), 21.4(-CH_3_). Compared with the data given in reference [27,28], fraction I corresponded to emodin.

Fraction II: EI-MS *m/z*: 254 [M]^+^; ^1^H-NMR (400 MHz, DMSO-*d*_6_) δ: 11.96 (1H, s, -OH), 11.86 (1H, s, -OH), 7.80 (1H, dd, *J* = 8.4, 7.6 Hz, H-6), 7.71 (1H, d, *J* = 7.6 Hz, H-5), 7.55 (1H, d, *J* = 0.8 Hz, H-4), 7.38 (1H, d, *J* = 8.4 Hz, H-7), 7.22 (1H, d, *J* = 0.8 Hz, H-2), 2.44 (3H, s, -CH_3_); ^13^C-NMR (100 MHz, DMSO-*d*_6_) δ: 191.4 (C-9), 181.3 (C-10), 161.4 (C-8), 161.1 (C-1), 149.0 (C-3), 137.2 (C-6), 133.2 (C-10a)，132.8 (C-4a), 124.2 (C-2), 123.9 (C-7), 120.4 (C-4), 119.2 (C-5), 115.7 (C-8a), 113.6 (C-9a)，21.6 (-CH_3_). Compared with the data given in reference [27,28], fraction II corresponded to chrysophanol.

Fraction III: EI-MS *m/z*: 284 [M]^+^; ^1^H-NMR (400 MHz, CDCl_3_) δ: 12.31 (1H, s, -OH), 12.11 (1H, s, -OH), 7.63 (1H, br s, H-4), 7.37 (1H, d, *J* = 2.4 Hz, H-5), 7.08 (1H, br s, H-2), 6.69 (1H, d, *J* = 2.4Hz, H-7), 3.94 (3H, s, -OCH_3_), 2.46 (3H, s, -CH_3_); ^13^C-NMR (100 MHz, CDCl_3_) δ: 190.7 (C-9), 181.9 (C-10), 166.5 (C-8), 165.1 (C-1), 162.4 (C-6), 148.4 (C-3), 135.2 (C-10a), 133.2 (C-4a), 124.4 (C-4), 121.2 (C-2), 113.7 (C-9a), 110.2 (C-8a), 108.2 (C-5), 106.8 (C-7), 56.1 (-OCH_3_), 22.2(-CH_3_). Compared with the data given in reference [27,28], fraction III corresponded to physcion. 

## 4. Conclusions

Three anthraquinones –emodin, chrysophanol, and physcion – were separated from the crude extract of *Rumex japonicus* by HSCCC for the first time. These compounds can be isolated on a sufficiently large scale with high purities which may then be used as reference substances for chromatography or for bioactivity studies. The method is a feasible, economical, and efficient technique for rapid preparative isolation of complicated natural products.
